# Disruption of STAT6 Signal Promotes Cardiac Fibrosis Through the Mobilization and Transformation of CD11b^+^ Immature Myeloid Cells

**DOI:** 10.3389/fphys.2020.579712

**Published:** 2020-10-22

**Authors:** Weiwei Zhang, Baoling Zhu, Suling Ding, Xiangfei Wang, Jian Wu, Xiaowei Zhu, Yunzeng Zou, Junbo Ge, Minghong Tong, Xiangdong Yang

**Affiliations:** ^1^Shanghai Institute of Cardiovascular Diseases, Zhongshan Hospital, Fudan University, Shanghai, China; ^2^Department of Cardiology, Zhongshan Hospital, Fudan University, Shanghai, China; ^3^Division of Clinical Laboratory, TongRen Hospital, Shanghai Jiao Tong University School of Medicine, Shanghai, China

**Keywords:** cardiac fibrosis, STAT6, isoproterenol, β-adrenergic receptor, inflammatory, CD11b^+^ myeloid cells, macrophages

## Abstract

Cardiac fibrosis is an important pathological basis of various cardiovascular diseases. The roles of STAT6 signal in allergy, immune regulation, tumorigenesis, and renal fibrosis have been documented. However, the function and mechanism of STAT6 signal in sympathetic overactivation-induced cardiac fibrosis have not been fully elucidated. This study explores the novel role of STAT6 signal in isoproterenol (ISO)–induced cardiac fibrosis through the regulation of inflammatory response and the differentiation of macrophages from immature myeloid cells. The expression levels of STAT6, β1-adrenergic receptor (β1-AR), and inflammatory factors [interleukin α (IL-1α), IL-6, IL-18, and transforming growth factor β (TGF-β)] in CD11b^+^ myeloid cells were analyzed with a microarray study. The levels of IL-6 and TGF-β1 in the CD11b^+^ myeloid cells–derived macrophages were detected with reverse transcriptase–polymerase chain reaction (RT-PCR). STAT6–knockout (KO) and WT mice were used to establish a murine cardiac fibrosis model by ISO injection. Cardiac fibroblasts were isolated from the hearts of newborn STAT6-KO and WT mice, and STAT6 expression was measured by Western blotting and RT-PCR after ISO stimulation, while α-smooth muscle actin (α-SMA) expression was detected by immunofluorescence and immunohistochemistry staining. Cardiac function and pathological characteristics were examined by echocardiography and immunohistochemistry staining, respectively. Immunohistochemistry staining with anti-CD11b was performed to detect the infiltration of CD11b^+^ myeloid cells in heart tissue. Flow cytometry analysis was used to measure the percentages of CD11b^+^ myeloid cells and CD11b^+^Ly6C^+^ macrophages in the peripheral blood. The results showed that STAT6 was highly expressed in CD11b^+^ myeloid cells located in injured hearts, and STAT6 expression in cardiac fibroblasts was down-regulated after ISO treatment. STAT6 deficiency further aggravated ISO-induced increased expression of α-SMA in cardiac fibroblasts, myocardial fibrosis, and cardiac dysfunction. The activation of ISO/β1-AR signal aggravated cardiac inflammatory infiltration, promoted CD11b^+^ myeloid cell mobilization, and enhanced CD11b^+^Ly6C^+/low^ macrophage differentiation, which was further exacerbated by STAT6 deficiency. Furthermore, β1-AR mRNA expression significantly increased in splenic CD11b^+^ myeloid cells compared to their bone marrow–derived controls, and STAT6 deficiency promoted β1-AR expression in an MI-induced sensitive cardiac fibrosis mouse model. The spleen-derived CD11b^+^ myeloid cells of STAT6-KO mice produced more IL-1α, IL-18, and TGF-β than their WT counterparts. Taken together, these results suggest that STAT6 signal plays a critical role in ISO-induced β1-AR overactivation and systemic inflammatory cascades, contributing to cardiac fibrogenesis. STAT6 should be a promising cardioprotective target against myocardial fibrosis and heart failure after β1-AR overactivation–induced myocardial injury.

## Introduction

The proliferation of fibroblasts and collagen secretion are crucial for the repair of cardiomyocytes after acute ischemic injury due to the poor regenerative abilities of cardiomyocytes. However, cardiac fibrosis post–acute myocardial ischemic injury is a double-edged sword. On the one hand, fibroblasts can repair the structural damage caused by myocardial necrosis. On the other hand, the excessive proliferation of myofibroblasts and the release of inflammatory cytokines aggravate the pathological remodeling of myocardium. It is well known that the pathological features of cardiac remodeling induced by hypertension are cardiomyocyte hypertrophy and cardiac interstitial fibrosis (Cuspidi et al., 2020; Ruan et al., 2018).

Chronic overactivation of the sympathetic nervous system by stimulation of β-adrenergic receptors (β-ARs) has been demonstrated to play a critical role in the development of hypertension and cardiac fibrosis (Alemasi et al., 2019), whereas cardiac sympathetic afferent denervation could suppress deleterious cardiac remodeling and improve cardiovascular dysfunction (Wang et al., 2014). By stimulating β-ARs, catecholamine overload leads to cardiac remodeling (Morishige et al., 2019). Isoproterenol (ISO) is a nonspecific β-AR agonist, and low-dose continuous injection of ISO may contribute to sustained β-AR stimulation in cardiomyocytes. A recent study revealed that acute β-AR overactivation by single-dose ISO injection could trigger interleukin 18 (IL-18)–dependent cytokine cascades, macrophage infiltration, and cardiac remodeling (Xiao et al., 2018). N-propargyl caffeate amide was reported to prevent cardiac fibrosis induced by myocardial infarction (MI) by enhancing pro-resolving macrophage polarization (Cheng et al., 2020). These studies highlight the critical role of innate immune cells in cardiovascular diseases caused by sympathetic dysfunction. However, more investigation is needed in order to understand the mechanisms behind this effect.

Histamine is a biogenic amine that has variable roles in allergies, inflammation, and gastric acid secretion and has also been linked to immune responses and tumorigenesis (Yang et al., 2011). Histidine decarboxylase (HDC) is the unique enzyme responsible for the conversion of L-histidine to histamine. Our previous studies demonstrated that histamine deficiency in HDC knockout (HDC-KO) mice could promote myocardial injury and cardiac fibrosis through aggravating macrophage dysfunction and cardiomyocyte apoptosis post-MI (Deng et al., 2015). The STAT6 signal was identified to mediate the effect of histamine on macrophage differentiation from CD11b^+^Gr-1^+^ immature myeloid cells (Xu et al., 2017). Furthermore, aggravated cardiac fibrogenesis was also examined in STAT6-KO mice (Chen et al., 2017). STAT6 has been found to be essential for the regulation of immune response and allergic response (Haase et al., 2020; Nam et al., 2020). Activation of the sympathetic nervous system has been demonstrated in acute MI (Graham et al., 2004; Hogarth et al., 2009; Hogarth et al., 2006). However, the roles and mechanisms of the β-AR signaling pathway in innate immune cell activation and myocardial fibrosis have not been fully elucidated.

The role of STAT6 and β-AR signals in cardiac fibrosis has not yet been characterized. To address this question, the expression levels of β1-AR mRNA were analyzed in CD11b^+^ myeloid cells directly isolated from the spleen and bone marrow of mice with MI-induced cardiac fibrosis. Furthermore, the activation of immune subsets in the peripheral blood and injured hearts of mice with ISO treatment was analyzed by flow cytometry analysis and histopathological study, respectively. STAT6-KO mice were also used to establish an ISO-induced cardiac fibrosis model and to investigate the effects of STAT6 deficiency on macrophage activation and fibroblast proliferation. We identified a higher level of β1-AR mRNA expressed in the spleen-derived CD11b^+^ myeloid cells compared to the bone marrow–derived counterparts. Overactivation of the β1-AR signal by ISO stimulation aggravated cardiac fibrosis mainly through the regulation of inflammatory infiltration, myeloid immune cell mobilization, and macrophage differentiation in STAT6 deficiency mice.

## Materials and Methods

### Animals

Male HDC-enhanced green fluorescent protein (EGFP), HDC-KO, STAT6-KO, and wild-type (WT) mice (Balb/C background, 10 weeks old) were purchased from the Model Animal Research Center of Nanjing University. All mice were housed in the SPF (specific pathogen–free) environment at 25°C with a 12-h bright/dark alternation and allowed free access to water and food. All animal experiments were reviewed and approved by the Animal Ethics Committee of Fudan University. Our study was performed in accordance with the Guidelines for the Care and Use of Laboratory Animals, which was published by the US National Institutes of Health (NIH Publication, 8th edition, 2011).

### ISO-Induced Cardiac Fibrosis Model Establishment

ISO (Sigma–Aldrich, United States) was formulated in saline prior to use. The mice were divided into six groups as follows: (i) WT + ISO 1 week: daily administration of ISO (5 mg/kg per day) for 1 week; (ii) WT + ISO 4 weeks: daily administration of ISO (5 mg/kg per day) for 4 weeks; (iii) WT control: daily subcutaneous administration of saline (same volume as ISO group) for 4 weeks; (iv) STAT6-KO + ISO 1 week: daily administration of ISO (5 mg/kg per day) for 1 week; (v) STAT6-KO + ISO 4 weeks: daily administration of ISO (5 mg/kg per day) for 4 weeks; (vi) STAT6-KO control: daily subcutaneous administration of saline for 4 weeks. Mice were euthanized approximately 24 h after the last injection.

### Echocardiography

The echocardiography was performed using a high-resolution small animal ultrasound system VisualSonics Vevo770 (VISUALSONIC, Canada). Mice were anesthetized under 2% isoflurane concentrations, and parastolic left ventricular long-axis view was captured. The following parameters of left ventricular ejection fraction (EF%) and left ventricular fractional shortening (FS%) were selected to evaluate cardiac function. EF% and FS% were calculated by the Simpson method as previously reported (Heinen et al., 2018). Each parameter took the mean value of three consecutive cardiac cycles.

### Flow Cytometry Analysis

Approximately 24 h after the last injection, mice were euthanized. The peripheral blood was collected and made into single-cell suspension. Erythrocyte lysate was used to get rid of red blood cells. Then cells were centrifuged and resuspended with 1 × phosphate-buffered saline (PBS). APC-CD11b (Biolegend, 101212), Gr-1-PerCP-Cy5.5 (BD, 552093), and Ly6C-PE (BD, 560592) fluorescent antibodies were used to incubate and stain cells for 45 min at 4°C. After washing, cells were resuspended with 300 μL PBS. The data were collected using a LSRII flow cytometer (BD Biosciences) and analyzed using FlowJo v7 software (Tree Star, Inc.).

### Histology and Immunohistochemistry Staining

After mice euthanasia, the heart was perfused with cold PBS and fixed with 10% neutral-buffered formalin at 4°C overnight. Then, the heart was embedded in paraffin and cut into 5-μm-thick coronal slides for further procedures. Sirius red staining and Masson staining were used to detect cardiac fibrosis; hematoxylin and eosin (HE) staining was used for morphology and inflammatory analysis, and immunohistochemistry staining was used to test the expression of α-SMA (Abcam, ab5694) and CD11b (BD, 553308) in heart tissues. The heart sections were captured with a light microscopy (Leica, Germany). Image-Pro-Plus 6 software was used to measure the area of anti-CD11b staining, collagen, and heart, respectively. The ratio of anti-CD11b staining area to heart area was used to evaluate cardiac CD11b^+^ infiltration; the ratio of collagen area to heart area was used to evaluate cardiac fibrosis.

### Cell Culture and Treatment

Primary cardiac fibroblasts were isolated from newborn (days 0–2) mice as described previously (Sawaki et al., 2018). Then, fibroblasts were collected and cultured in Dulbecco modified eagle medium supplemented with 10% fetal bovine serum (Sigma–Aldrich, United States) and 1% penicillin-streptomycin in a humidified environment at 37°C with 5% CO_2_. Generation 2–4 fibroblasts were used in this study. Fibroblasts were cultured at densities of less than 80% confluency in a six-well culture plate, and the serum-free medium was used for at least 24 h before ISO treatment. Then the cells were stimulated by ISO and harvested after 24 h of continuous stimulation. In control groups, fibroblasts were given an equal volume of PBS instead. The mRNA and protein expression were measured with polymerase chain reaction (PCR) and Western blotting, respectively.

Bone marrow–derived cells were isolated from the femur bones of WT and STAT6-KO mice and made into single-cell suspensions, and the FACS lysing solution (BD) was used to lyse red blood cells. CD11b^+^ cells were then sorted by magnetic beads. CD11b^+^ cells were cultured in six-well culture plates (approximately 2.5 million cells/well) with RPMI-1640 medium (Hyclone) supplemented with 10% fetal bovine serum (Sigma–Aldrich, United StatesA) and 1% penicillin-streptomycin in a humidified environment (37°C at 5% CO_2_ concentration). The cells were induced to differentiate into macrophages with macrophage colony-stimulating factor (M-CSF, PeproTech, 100 ng/mL) for 5–7 days, and the liquid medium was changed every 2 days. After the last change of the culture medium, the macrophages were cultured in serum-free medium for 24 h. Then, the macrophages were stimulated with ISO for 15 min and collected with Trizol for the subsequent detection of inflammatory factors mRNA expression.

### Immunofluorescence Staining

Cardiac fibroblasts were isolated from STAT6-KO and WT mice hearts and cultured as above. Cardiac fibroblasts were fixed with 4% (vol/vol) paraformaldehyde for 20 min, permeabilized with 0.2% TritonX-100 for 20 min, and blocked with 5% bovine serum albumin for 1 h at room temperature. Then samples were incubated with anti–α-SMA antibody (Abcam, ab5694) at 1:200 overnight at 4°C. After washing three times with 1 × PBS, cells were incubated with Alexa Flour 594–conjugated donkey anti–rabbit immunoglobulin G (1:400) for 1 h at room temperature in a dark environment. After washing, DAPI (Beyotime Biotechnology, China) was used to stain nuclei. The method of immunofluorescence for frozen section was similar to our previous study (Chen et al., 2017). The immunofluorescence staining method for heart and spleen tissues is as similar as above. All photographs were taken with a fluorescence microscope (Leica, Germany).

### Quantitative Real-Time PCR

Total RNA was extracted by TRIzol reagent (Invitrogen, Waltham, MA, United States) for mRNA quantification. Then 1 μg of RNA was reverse transcribed with a PrimeScript RT reagent Kit with gDNA Eraser (Takara, Japan). The gene expression level of STAT6 was quantified by SYBR Premix Ex Taq (Takara, Japan). Data analysis was performed with an Applied Biosystems Prism7500 sequence detection system. Based on the sequences that were available in NCBI database, gene primers of β-actin, GAPDH, STAT6, IL-6, and transforming growth factor β (TGF-β) were designed by Sangon Biotech (Shanghai, China), which is shown in detail in the Table 1. After the expression of β-actin or GAPDH was normalized, the fold change of STAT6, IL-6, and TGF-β1 expression was calculated according to the ΔΔ cycle time (Ct) method.

**TABLE 1:** Primers for RT-PCR.

Gene	Forward primer	Reverse primer
β-Actin	ACGTTGACATCCGTAAAGACC	ACACAGAGTACTTGCGCTCA
GAPDH	GACATCAAGAAGGTGGTGAAGCAG	ATACCAG GAAATG AGCTTG ACAAA
STAT6	CTCTGTGG G GCCTAATTTCCA	GCATCTGAACCGACCAGGAAC
IL-6	CCAAGAGGTGAGTGCTTCCC	CTGTTGTTCAGACTCTCTCCCT
TGF-βl	TCTGCATTGCACTTATGCTGA	AAAGGGCGATCTAGTGATGGA

### Western Blotting

Cardiac fibroblasts were lysed on ice with RIPA lysis buffer (Beyotime Biotechnology, China) supplemented with protease and phosphatase inhibitors for protein extraction. The protein concentrations were measured by BCA protein assay kit (Beyotime Biotechnology, China). Proteins were loaded onto 10% sodium dodecyl sulfate–polyacrylamide gel electrophoresis and were blotted onto the polyvinylidene fluoride membrane. These membranes were incubated with 5% nonfat dry milk to block proteins for 1 h. Next, membranes were incubated and stained with primary antibodies overnight at 4°C. The primary antibodies were diluted for 1:1,000 and 1:5,000 for anti-STAT6 (Abcam, ab44718) and anti-GAPDH (Abcam, ab8245), respectively. On the next day, membranes were washed with 1 × TBST (Tris-buffered saline + Tween 20) for three times and were then incubated with second antibodies labeled by horseradish peroxidase for 2 h. Membranes were washed with 1 × TBST for three times and were quantified by scanning densitometries.

### Microarray Performance and Analysis

Differentially expressed genes of CD11b^+^ myeloid cells in the spleen and bone marrow of WT, HDC-KO, and STAT6-KO mice (five mice in each group) were examined by microarray analysis (Agilent Mouse 4x44K Gene Expression Microarrays). Total RNA was extracted from CD11b^+^ cells (10 million cells) isolated from the spleen and bone marrow of MI and control mice for microarray studies. The sample preparation and chip data analysis of Gene Expression Microarrays were described in detail in our previous article (Xu et al., 2017). The microarray data have been deposited in the GEO (Gene Expression Omnibus) database (accession number: GSE154733). Basic analysis was performed with Genespring software. Then, according to the threshold (fold change >2.0 and *P* < 0.05), differentially expressed genes were selected out.

### Statistical Methods

GraphPad Prism software was used for statistical analysis, and data were expressed with *$\bar{x}\pm s$*. Student *t* test was used to compare data between two groups, and one-way analysis of variance (ANOVA) was used to compare data among multiple groups. When two factors were involved, data were statistically analyzed by the two-way ANOVA analysis. *P* < 0.05 was considered to be statistically significant.

## Results

### β1-AR Expression Increased in the Splenic CD11b^+^ Myeloid Cells in HDC-KO Mice With MI

**FIGURE 1:**
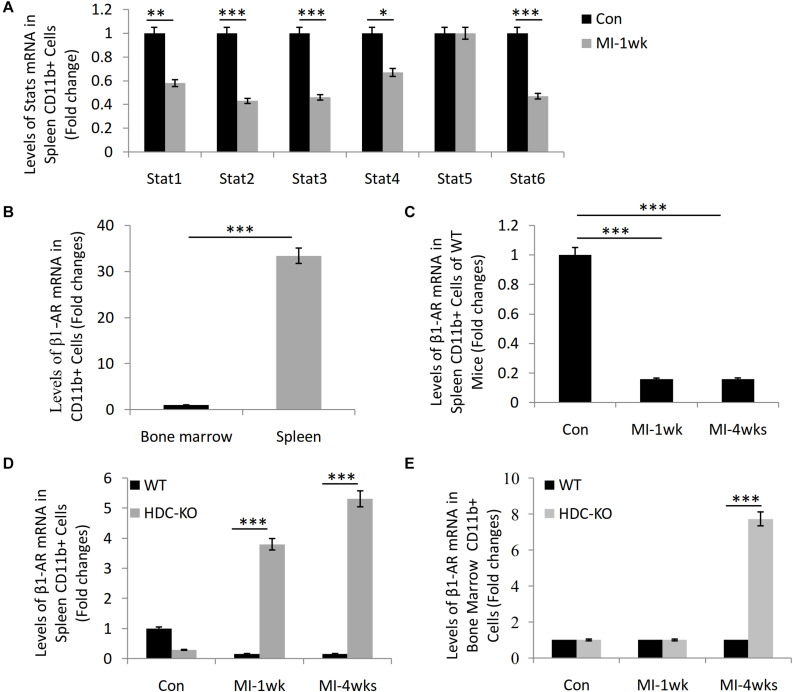
Microarray study data showing STAT6 and β1-AR expression in CD11b^+^ myeloid cells of mice with MI. **(A)** The mRNA levels of STAT1, STAT2, STAT3, STAT4, STAT5, and STAT6 in the splenic CD11b^+^ cells isolated from WT mice 1 week after MI. **(B)** The expression of β1-AR in bone marrow and spleen-derived CD11b^+^ cells of WT mice. **(C)** The expression of β1-AR in spleen-derived CD11b^+^ cells of WT mice after MI. **(D)** The expression of β1-AR in spleen-derived CD11b^+^ cells of HDC-KO and WT mice after MI. **(E)** The expression of β1-AR in bone marrow–derived CD11b^+^ cells of HDC-KO and WT mice after MI. ***P < 0.001, **P < 0.01, *P < 0.05. *n* = 5 per group.

Our previous studies have shown that HDC-KO mice are sensitive models of cardiac fibrosis after MI and that histamine deficiency inhibits the expression of STAT6 signal in CD11b^+^Ly6C^+^ macrophages (Xu et al., 2017; Chen et al., 2017). By microarray analysis, the data showed the downregulation of STAT1, STAT2, STAT3, STAT4, and STAT6 mRNA in the spleen-derived CD11b^+^ cells after MI-1wk compared to sham group (Figure 1A). Furthermore, we also observed a higher level expression of β1-AR mRNA in the spleen-derived CD11b^+^ cells than that of bone marrow–derived CD11b^+^ cells (Figure 1B). The expression of β1-AR decreased in the spleen-derived CD11b^+^ cells of WT mice both 1 week and 4 weeks after MI (Figure 1C). Interestingly, we found significant upregulation of β1-AR in the spleen-derived CD11b^+^ cells of HDC-KO mice at 1 week and 4 weeks after MI compared to WT mice (Figure 1D). A similar result was examined in the bone marrow–derived CD11b^+^ cells of HDC-KO mice at 4 weeks after MI compared to WT mice (Figure 1E). Taken together, these data not only confirm that the spleen is the main cellular source of CD11b^+^ myeloid cells in the early stage of MI, but also suggest that the β-AR–related sympathetic signal and the STAT6 signal are involved in the activation of CD11b^+^ immune cells and cardiac fibrogenesis after MI.

### STAT6 Was Highly Expressed in CD11b^+^ Myeloid Cells Infiltrated Into Mouse Heart

**FIGURE 2:**
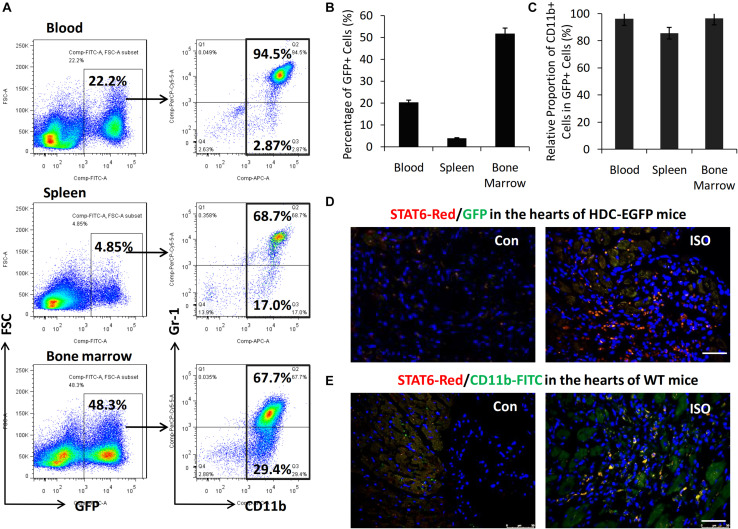
High level expression of STAT6 in CD11b^+^ myeloid cells in mouse heart. **(A)** Representative FACS graphs show the percentage of CD11b^+^GFP^+^ cells in the blood, spleen, and bone marrow of HDC-EGFP mice. **(B)** Quantitative analysis of percentage of GFP^+^ cells in the blood, spleen, and bone marrow of HDC-EGFP mice. **(C)** Quantitative analysis of the relative proportion of CD11b^+^ myeloid cells in total GFP^+^ cells in the blood, spleen, and bone marrow of HDC-EGFP mice. **(D)** The expression of STAT6 (red) in the injured hearts of HDC-EGFP mice after ISO stimulation. Scale bar, 50 μm. **(E)** The expression of STAT6 (red) its colocalization with CD11b^+^ (green). Scale bar, 50 μm. *n* = 5–6 per group.

HDC-EGFP transgenic mice provide a very convenient tool for tracing GFP-expressing CD11b^+^ myeloid immune cells *in vivo* (Yang et al., 2011). FACS data showed that about 40–50% of bone marrow cells, 3–5% of spleen cells, and 10% to 20% of blood cells were GFP^+^ and thus expressing HDC (Figures 2A,B). Furthermore, FACS data revealed that the majority of GFP^+^ cells expressed myeloid cell marker CD11b (90–95%) including CD11b^+^Gr-1^+^ granulocytic cells and CD11b^+^Gr-1^–^ monocytic cells (Figures 2A,C). In this study, we examined the expression of GFP and STAT6 in the hearts of HDC-EGFP mice with ISO treatment (three times I.P). The results of immunostaining with anti-STAT6 showed that the expression levels of STAT6 and GFP-expressing myeloid cells increased in the injured hearts of HDC-EGFP mice after ISO stimulation; the colocalization results showed that STAT6 was highly expressed in GFP^+^ myeloid cells and cardiac interstitial cells instead of cardiomyocytes (Figure 2D). Furthermore, the result of coimmunostaining with anti-STAT6 (red) and anti-CD11b (green) confirmed a high level expression of STAT6 protein in CD11b^+^ myeloid cells (Figure 2E). Spleen tissue was used as a positive control because of high level expression of STAT6 in lymphocytes and CD11b^+^ cells (data not shown). Thus, these results suggest that the STAT6 signaling pathway is involved in the expression and function of CD11b^+^ myeloid cells in stress-induced myocardial injury and repair.

### STAT6 Expression Decreased in ISO-Treated Fibroblasts and STAT6 Deficiency Promoted Cardiac Fibroblast Transdifferentiation

**FIGURE 3:**
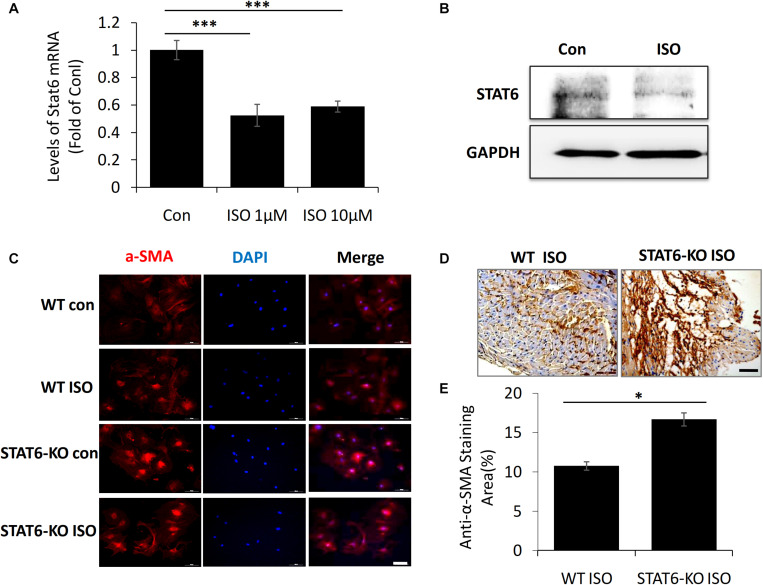
The expression of STAT6 and α-SMA expression in cardiac fibroblasts upon ISO stimulation. **(A)** The mRNA level of STAT6 expression by RT-PCR (*n* = 3). **(B)** The protein level of STAT6 expression by Western blotting. **(C)** Representative images of immunofluorescence staining for α-SMA in isolated cardiac fibroblasts upon ISO treatment. Scale bar, 100 μm, *n* = 3. **(D)** Representative images of immunohistochemical staining for α-SMA in heart tissue after ISO treatment for 4 weeks. Scale bar, 50 μm, *n* = 6. **(E)** Quantitative analysis of anti–α-SMA area (%) with immunohistochemical staining in heart tissue after ISO treatment for 4 weeks (*n* = 6). ***P < 0.001, *P < 0.05.

To further investigate the link between ISO and STAT6 expression, we isolated and cultured the cardiac fibroblasts from neonatal mice *in vitro*. The result of quantitative PCR showed that STAT6 mRNA expression was significantly downregulated in cardiac fibroblasts following ISO treatment (Figure 3A). Western blotting data further confirmed that ISO remarkably reduced the expression of STAT6 protein (Figure 3B).

Next, to investigate the role of STAT6 in ISO-induced cardiac myofibroblasts transdifferentiation, we stained α-SMA (a canonical marker of myofibroblasts) *in vivo* and *in vitro*. Immunofluorescence staining with anti–α-SMA showed that the expression of α-SMA in cardiac fibroblasts was significantly up-regulated upon ISO stimulation, and more α-SMA^+^ myofibroblasts proliferated and accumulated in the hearts of STAT6-KO mice compared with that of WT mice (Figure 3C). The expression of α-SMA in cardiac tissue was detected by immunohistochemical staining. The results confirmed that the hearts of STAT6-KO mice exhibited abundant positively α-SMA–labeled cells than that of WT mice by ISO administration (Figures 3D,E). These data demonstrate that the STAT6 signaling pathway has a close relationship with ISO-induced cardiac fibrosis, and STAT6 deficiency aggravates ISO-induced cardiac α-SMA^+^ myofibroblasts differentiation and proliferation in mice.

### STAT6 Deficiency Aggravated ISO-Induced Cardiac Dysfunction and Fibrosis in Mice

**FIGURE 4:**
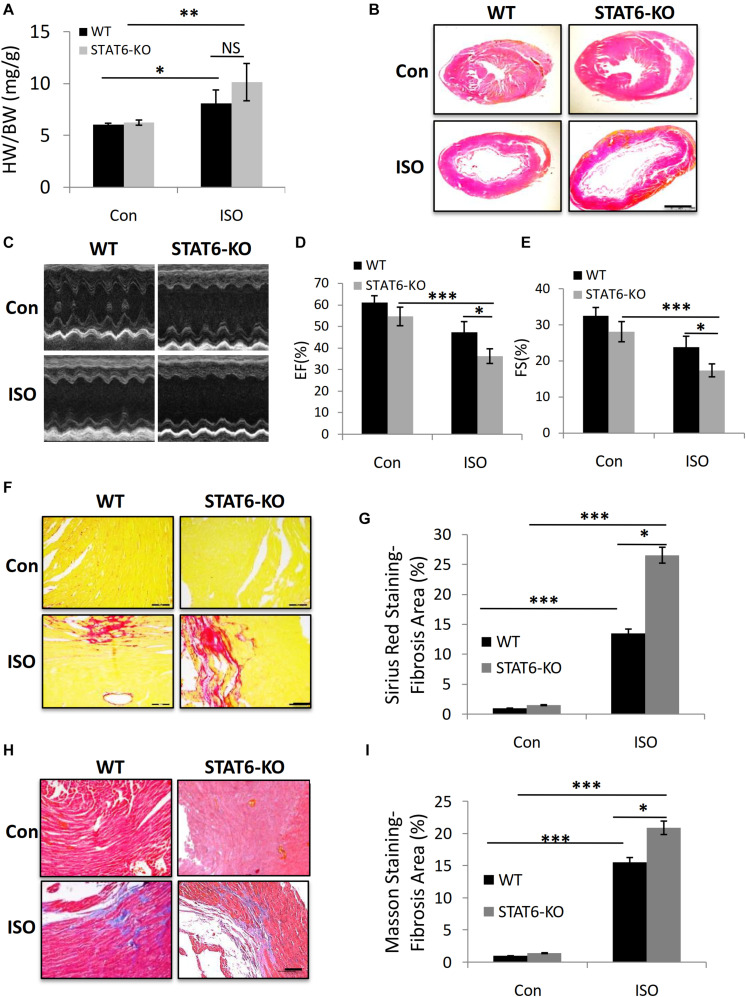
The impact of STAT6 deficiency on ISO-induced cardiac dysfunction and fibrosis in mice. **(A)** The HW/BW ratio of mice. **(B)** Representative HE staining images of heart. Scale bar, 1,000 μm. **(C)** Representative parasternal long-axis view echocardiographic M-mode images after ISO treatment for 4 weeks. **(D)** Quantitative analysis of EF (%) after ISO treatment for 4 weeks. **(E)** Quantitative analysis of FS (%) after ISO treatment for 4 weeks. **(F)** Representative micrographs of Sirius red staining. Scale bar, 100 μm. **(G)** Quantitative analysis of fibrosis area (%) with Sirius red staining. **(H)** Representative micrographs of Masson staining. Scale bar, 100 μm. **(I)** Quantitative analysis of fibrosis area (%) with Masson staining. ***P < 0.001, **P < 0.01, *P < 0.05. *n* = 6–8 per group.

We sought to explore the role of STAT6 signal in ISO-induced cardiac fibrosis. Age- and gender-matched STAT6 and WT mice were treated with ISO injection for 4 weeks. The ratio of heart weight to body weight (HW/BW) increased in STAT6-KO and WT mice with ISO compared to control groups; a significant upward shift of the HW/BW ratio was examined in ISO-treated STAT6-KO mice compared to ISO-treated WT mice (Figure 4A). Furthermore, HE staining data showed that the ventricular cavity was enlarged in response to persistent ISO stimulation and that the hearts of STAT6-KO + ISO mice were significantly bigger than those of WT + ISO mice (Figure 4B). Echocardiography was performed to evaluate the left ventricular systolic function (Figure 4C). Cardiac function parameters (EF% and FS%) of ISO mice were significantly lower than those of control mice, reflecting the impairment of cardiac function. What’s more, EF% and FS% of STAT6-KO + ISO mice were significantly lower than those of WT + ISO mice (Figures 4D,E). The results of Sirius red staining (Figures 4F,G) and Masson staining (Figures 4H,I) showed that the hearts of ISO mice produced more interstitial collagen than those of control mice and that STAT6 deficiency significantly increased collagen accumulation after ISO stimulation compared to WT controls. Taken together, these data demonstrate that the STAT6 signal plays a critical role in ISO-induced cardiac dysfunction and that its deficiency promotes cardiac fibrosis.

### STAT6 Knockout Promoted CD11b^+^ Myeloid Cells Mobilization, CD11b^+^Ly6C^+^ Macrophage Differentiation, and Aggravated ISO-Induced Cardiac Inflammation

**FIGURE 5:**
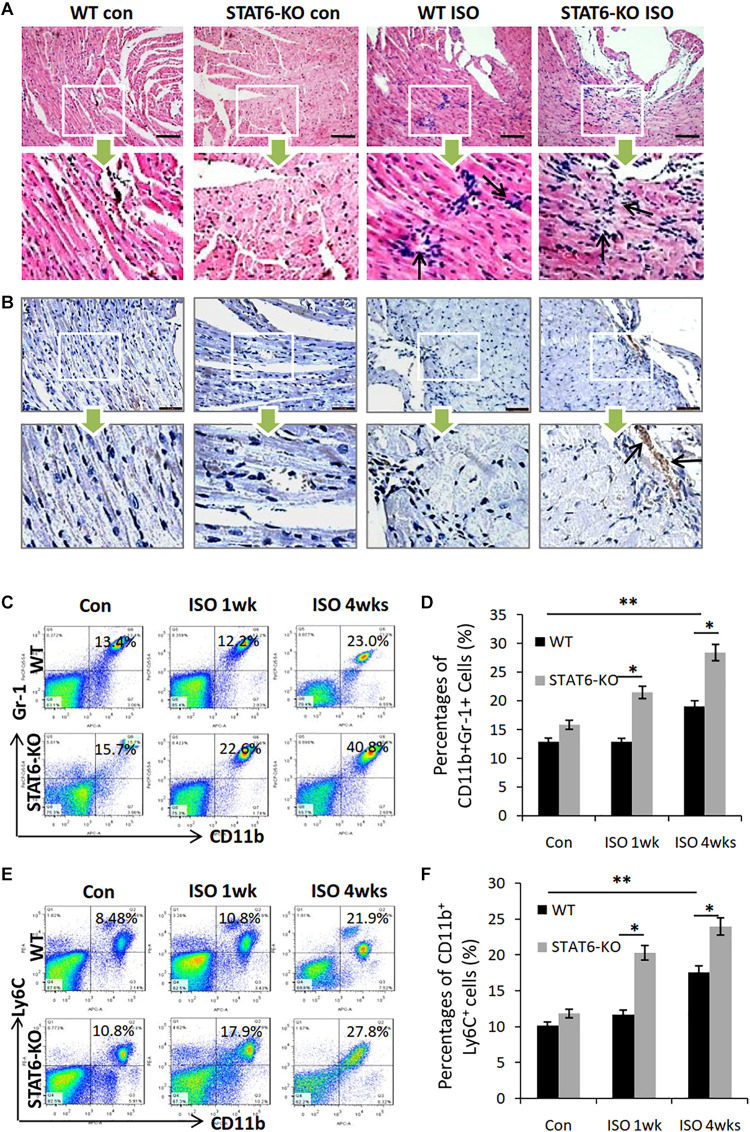
The impact of ISO and STAT6 signal on systemic and cardiac inflammatory responses. **(A)** Representative HE staining images of heart sections, and the black arrows refer to the infiltrated inflammatory cells. Scale bar, 100 μm. **(B)** Representative images of immunohistochemical staining for CD11b in hearts, and the black arrows refer to the infiltrated CD11b^+^ cells. Scale bar, 50 μm. **(C)** Representative FACS graphs show the percentage of CD11b^+^Gr-1^+^ myeloid cells following ISO treatment. **(D)** Quantitative analysis of percentage of CD11b^+^Gr-1^+^ myeloid cells. **(E)** Representative FACS graphs show the percentage of CD11b^+^Ly6C^+^ myeloid cells following ISO treatment. **(F)** Quantitative analysis of percentage of CD11b^+^Ly6C^+^ macrophages. ***P < 0.001, **P < 0.01, *P < 0.05. *n* = 6–8 per group.

Given that ISO treatment could induce inflammatory reaction in the hearts of mice, we sought to investigate the effects of STAT6-KO on the expression of immune cells in the development of ISO-induced cardiac fibrosis. HE staining showed that the number of infiltrated immune cells significantly increased in the hearts of STAT6-KO + ISO mice compared to those of WT + ISO mice; concurrently, we also found that STAT6-KO did not significantly increase the immune cells in the hearts without ISO stimulation (Figure 5A). We further examined the expression of CD11b in cardiac tissue by immunohistochemical staining, which showed that STAT6-KO significantly increased the infiltration of CD11b^+^ myeloid cells in the injured hearts caused by ISO (Figure 5B). What’s more, FACS data showed that after ISO treatment, the percentage of CD11b^+^Gr1^+^ myeloid cells increased in the peripheral blood of STAT6-KO mice compared to WT mice with ISO treatment (Figures 5C,D). Additionally, FACS data showed that the percentage of CD11b^+^ly6C^+^ monocytes/macrophages increased in the peripheral blood of STAT6-KO + ISO mice than that of WT + ISO mice (Figures 5E,F). These results suggest that STAT6 deficiency mediates a crucial role in ISO-induced inflammatory storm, which is characterized by aggravating cardiac inflammatory infiltration, increasing CD11b^+^ myeloid cell mobilization, and promoting CD11b^+^ly6C^+^ macrophage differentiation.

### STAT6 Deficiency Promoted the Expression of β1-AR and Inflammatory Factors in Splenic CD11b^+^ Cells and Myeloid Cell–Derived Macrophages

**FIGURE 6:**
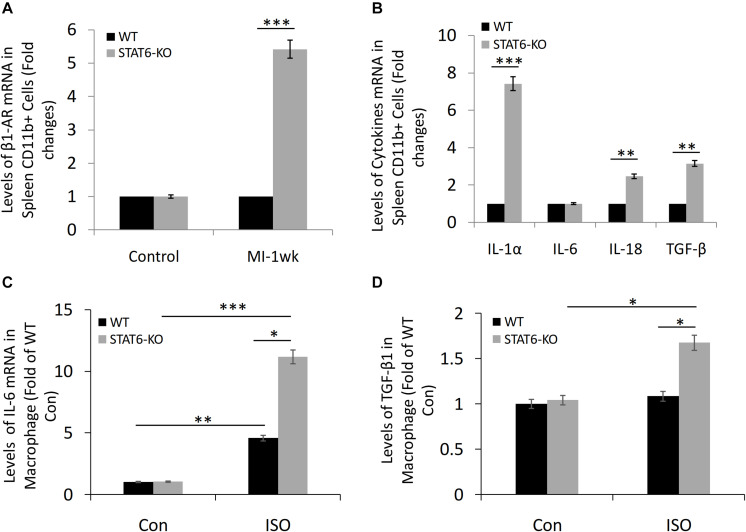
The expression of β1-AR, IL-1α, IL-6, IL-18, and TGF-β in CD11b^+^ cells and macrophages of STAT6-KO and WT mice. **(A)** Microarray study data showing the expression of β1-AR in spleen-derived CD11b^+^ cells of STAT6-KO and WT mice after MI (*n* = 5 per group). **(B)** Microarray study data showing the expression of inflammatory factors in spleen-derived CD11b^+^ cells of STAT6-KO and WT mice (*n* = 5 per group). **(C)** The mRNA level of IL-6 expression in the CD11b^+^ myeloid cell–derived macrophages by RT-PCR (*n* = 3). **(D)** The mRNA level of TGF-β1 expression in the CD11b^+^ myeloid cell–derived macrophages by RT-PCR (*n* = 3). ^∗∗∗^*P* < 0.001, ^∗∗^*P* < 0.01, and ^∗^*P* < 0.05.

The microarray data revealed that the level of β1-AR expression in the spleen-derived CD11b^+^ cells of STAT6-KO mice was significantly higher than that of WT mice in a sensitive myocardial fibrosis model induced by MI (Figure 6A). Furthermore, we analyzed the effect of STAT6-KO on the expression of a series of inflammatory factors (IL-1α, IL-6, IL-18, and TGF-β), which had been proved to promote inflammation and participate in cytokine storms. Results showed that the expression levels of IL-1α, IL-18, and TGF-β in STAT6-KO splenic CD11b^+^ cells were significantly higher than their WT counterparts, while there was no significant difference in IL-6 expression between the two genotypes of splenic CD11b^+^ cells (Figure 6B). Besides, the bone marrow cells were isolated from STAT6-KO and WT mice and induced macrophage differentiation. The result of reverse transcriptase (RT)–PCR showed that STAT6-KO significantly increased the mRNA expression of IL-6 and TGF-β1 in macrophages after ISO treatment (Figures 6C,D). Thus, these results imply that STAT6 deficiency promotes ISO-induced cardiac fibrosis associated with the upregulation of β1-AR in splenic CD11b^+^ cells and the increasing of inflammatory cytokines in CD11b^+^ myeloid cells and macrophages.

## Discussion

In the present study, we show the first evidence that the disruption of the STAT6 signal aggravates ISO-induced adverse cardiac fibrosis, which mechanisms involve a series of events, including cardiomyocyte injury, β1-AR expression up-regulation, increased CD11b^+^ myeloid cell mobilization, increased macrophages differentiation, and collagen deposition aggravation in response to ISO stimulation. Taken together, these results provide novel evidence that STAT6 signal is a potential target for the treatment of myocardial fibrosis and heart failure after ISO-induced myocardial injury.

The sympathetic–catecholamine system is involved in the regulation of multiple cardiovascular physiological activities through adrenergic receptor (Winter et al., 2018; Schinner et al., 2017). β-AR is a member of the superfamily of G protein–coupled receptors, which are also called seven-transmembrane receptors (Vassilatis et al., 2003). There are three β-AR subtypes, β1-AR, β2-AR, and β3-AR. Among them, β1-AR is the predominant subtype of adrenergic receptor in heart tissue, which plays a pivotal role in mediating cardiac function (Chottova and Slavikova, 2011). After β1-AR activation, the classical Gs-AC-cAMP-PKA signaling cascade is activated. It is also noteworthy that β1-AR is closely related to excessive sympathetic stimulation and cardiac fibrosis. Previous studies have indicated that β1-AR transgenic overexpression mice show increased cardiac interstitial fibrosis, and altered calcium handling is responsible for these detrimental effects of β1-AR signaling (Engelhardt et al., 2004). Accumulating evidences have indicated that ISO, a nonselective β-AR agonist, can induce cardiac hypertrophy and fibrosis by activating multiple signaling pathways to regulate the expression of extracellular matrix, oxidative stress response, and inflammatory response and ultimately affect cardiac function (Tang et al., 2018; Wang M. et al., 2019). In this study, we provide a new perspective to explain the effect of ISO on myocardial fibrogenesis. By a microarray study, we identified higher-level expression of β1-AR mRNA in CD11b^+^ myeloid cells directly isolated from the spleen, main extramedullary immune cell reservoir in the development of cardiac fibrosis of WT mice post-MI. Using the established HDC-KO mouse model sensitive to myocardial fibrosis, we further confirmed the increased expression of β1-AR mRNA in spleen and bone marrow–derived CD11b^+^ cells from HDC-KO mice compared to WT controls.

STAT6 signal has previously been demonstrated to mediate the effects of histamine on the protection of MI-induced myocardial injury and on the differentiation of macrophages from bone marrow–derived CD11b^+^ myeloid cells with granulocyte M-CSF incubation (Xu et al., 2017; Chen et al., 2017). Thus, these data highlight that STAT6 signaling pathway plays a key role in the regulation of inflammatory immune response in ischemic myocardial injury and fibrosis. However, the roles of STAT6 signal in myocardial injury and fibrosis caused by ISO/β1-AR–induced sympathetic nervous system hyperactivity are not fully clarified.

The STAT family consists of seven members (STAT1, 2, 3, 4, 5A, 5B, and 6) in mammals, which exists in the cytoplasm and can be transported into the nucleus after activation and binds to DNA (Wang and Levy, 2012). Among them, STAT6 plays a central role in immune regulation, cell proliferation, and apoptosis (Wang J. et al., 2019; Wang L. et al., 2019). In the current study, we confirmed that STAT6 was highly expressed in CD11b^+^ myeloid cells and cardiac fibroblasts than in cardiomyocytes. We found STAT6 deficiency increased β1-AR expression and aggravated ISO-induced cardiac fibrosis, with abnormal immune response, consistent with our previous study that STAT6 deficiency promotes the proliferation and differentiation of CD11b^+^Gr-1^+^ immature myeloid cells in the blood and bone marrow of MI mice. CD11b^+^Gr-1^+^ immature myeloid cells consist of CD11b^+^Ly6G^+^ granulocytic subset and CD11b^+^Ly6C^+^ monocytic subset. A previous study indicated that the expression of STAT6 signal was down-regulated during the differentiation of tumor-associated neutrophils from CD11b^+^Gr-1^+^ IMCs of HDC-KO mice (Yang et al., 2011). In this study, we found that STAT6 deficiency promoted the mobilization of CD11b^+^ IMCs and CD11b^+^Ly6C^+^ macrophage differentiation upon β-AR stimulation. Macrophages, as one of the most important antigen-presenting cells, play a pivotal role in the development of cardiac fibrosis. Macrophages potentially lead to angiotensin II–induced cardiac fibrosis when recruited into the heart from the spleen (Wang et al., 2017). Further, cardiac macrophages are responsible for impaired myocardial relaxation and aggravated myocardial stiffness while diastolic dysfunction develops (Hulsmans et al., 2018). It is known that β-ARs are expressed on the surface of macrophages in most tissues, and macrophage mechanotype and function can be regulated through β-AR by stress hormone signal (Kim et al., 2019). In this study, the data demonstrated that STAT6 deficiency promotes macrophage differentiation from CD11b^+^ IMCs and recruits them into the injured hearts after chronic stimulation of β-AR, which in turn aggravated cardiac fibrosis. Moreover, the microarray data showed that STAT6 gene knockout (KO) leads to an significant upsurge of a series of key cytokines (IL-1α, IL-18, and TGF-β), which served an important role in cytokine storm and immunopathological damage process (Huang et al., 2005; Yao et al., 2020). Furthermore, the expression of IL-6 and TGF-β1 in the CD11b^+^ myeloid cell–derived macrophages increased significantly with ISO stimulation, which could be further promoted by STAT6 deficiency.

In clinical settings, hypertensive heart diseases are major health problems worldwide and possess cardiac fibrosis that significantly reduces cardiac function. It is noteworthy that β-blockers are widely used to improve cardiac function in the failing heart and reverse cardiac remodeling (Cleland et al., 2018). However, besides mediating pathological cardiac remodeling, β-AR signal pathways also take part in normal cardiac physiological activity, which consists of positive chronotropic, dromotropic, and inotropic effects. So when β-blockers alleviate the pathological function, it also antagonizes the normal physiological effects, which is harmful to patients. Over the past years, more and more evidence has accumulated indicating that long-acting β-blockers can lead to some detrimental effects (Woron et al., 2019). In addition to β-blockers, anti–TGF-β antibodies have also been considered as potential antifibrotic agents (Kuwahara et al., 2002). Nevertheless, it is found that anti–TGF-β administration before or after coronary artery ligation contributes to increased mortality and worsened left ventricular remodeling in mice with MI, and ECM remodeling may be related to these detrimental effects (Frantz et al., 2008). Therefore, rationally designed antifibrotic therapies need to be explored and discovered to curb these problems. In this regard, STAT6 could confer cardioprotection in β-AR–mediated cardiac fibrosis chronically stimulated with catecholamine. As a novel antifibrotic strategy, STAT6 signal target does not interfere and damage the normal physiological function of β-AR, and no increased mortality has been found in mice. Thus, STAT6 signal could be a safe, promising, and viable candidate drug target against ISO-induced cardiac fibrosis.

## Data Availability Statement

The microarray data has been deposited in the GEO (Gene Expression Omnibus) database (accession number: GSE154733).

## Ethics Statement

The animal study was reviewed and approved by Animal Ethics Committee of Fudan University.

## Author Contributions

WZ, BZ, MT, and XY participated in the study design, experiment operation, and manuscript preparation. SD and XZ contributed to the animal experiments and statistical analysis. XW and JW helped with the collection and analysis of echocardiography. YZ and JG provided the suggestions for study design and helped with revision of the manuscript. All authors discussed and approved the manuscript.

## Conflict of Interest

The authors declare that the research was conducted in the absence of any commercial or financial relationships that could be construed as a potential conflict of interest.
